# IGF-1R mediates crosstalk between nasopharyngeal carcinoma cells and osteoclasts and promotes tumor bone metastasis

**DOI:** 10.1186/s13046-024-02970-8

**Published:** 2024-02-12

**Authors:** Kaifan Yang, Yanjun Hu, Yuanyuan Feng, Kaiqun Li, Ziyan Zhu, Shuyi Liu, Yanling Lin, Bin Yu

**Affiliations:** 1 Division of Orthopaedics and Traumatology, Department of Orthopaedics, Nanfang Hospital, Southern Medical University, Guangzhou, China; 2 Guangdong Provincial Key Laboratory of Bone and Cartilage Regenerative Medicine, Nanfang Hospital, Southern Medical University, Guangzhou, China; 3 Department of Radiation Oncology, Nanfang Hospital, Southern Medical University, Guangzhou, China; 4 The First School of Clinical Medicine, Southern Medical University, Guangzhou, China

**Keywords:** Nasopharyngeal carcinoma, Bone metastasis, IGF-1R, Osteoclasts, GM-CSF

## Abstract

**Background:**

Nasopharyngeal carcinoma (NPC) poses a significant health burden in specific regions of Asia, and some of NPC patients have bone metastases at the time of initial diagnosis. Bone metastasis can cause pathologic fractures and pain, reducing patients' quality of life, and is associated with worse survival. This study aims to unravel the complex role of insulin-like growth factor 1 receptor (IGF-1R) in NPC bone metastasis, offering insights into potential therapeutic targets.

**Methods:**

We assessed IGF-1R expression in NPC cells and explored its correlation with bone metastasis. Experiments investigated the impact of osteoclast-secreted IGF-1 on the IGF-1R/AKT/S6 pathway in promoting NPC cell proliferation within the bone marrow. Additionally, the reciprocal influence of tumor-secreted Granulocyte–macrophage colony-stimulating factor (GM-CSF) on osteoclast differentiation and bone resorption was examined. The effects of IGF-1 neutralizing antibody, IGF-1R specific inhibitor (NVP-AEW541) and mTORC inhibitor (rapamycin) on nasopharyngeal carcinoma bone metastasis were also explored in animal experiments.

**Results:**

Elevated IGF-1R expression in NPC cells correlated with an increased tendency for bone metastasis. IGF-1, secreted by osteoclasts, activated the IGF-1R/AKT/S6 pathway, promoting NPC cell proliferation in the bone marrow. Tumor-secreted GM-CSF further stimulated osteoclast differentiation, exacerbating bone resorption. The IGF-1 neutralizing antibody, NVP-AEW541 and rapamycin were respectively effective in slowing down the rate of bone metastasis and reducing bone destruction.

**Conclusion:**

The intricate interplay among IGF-1R, IGF-1, and GM-CSF highlights potential therapeutic targets for precise control of NPC bone metastasis, providing valuable insights for developing targeted interventions.

**Supplementary Information:**

The online version contains supplementary material available at 10.1186/s13046-024-02970-8.

## Background

Nasopharyngeal carcinoma is a common malignancy originating from epithelial tissue with a distinct regional distribution, commonly found in certain regions of East and Southeast Asia, such as southern China [1]. Although early-stage nasopharyngeal carcinoma can be cured with 5-year overall survival (OS) rates exceeding 90% [2, 3], patients are usually in advanced stage at the time of initial diagnosis, and about 15% of patients with nasopharyngeal carcinoma have distant metastases at the time of initial diagnosis [4], and the median overall survival of such patients is about 10–36 months [5–7]. Clearly, distant metastases are associated with worse survival. The bone is the most common site of metastasis, with an incidence of approximately 64%-67%, and tumor bone metastasis leads to the occurrence of multiple skeletal complications such as pathological fractures and bone pain, which greatly affect patients' quality of life and reduce survival [8, 9]. Therefore, it is important to predict and screen patients at high risk of nasopharyngeal carcinoma bone metastasis and provide them with precise treatment strategies to improve quality of life and survival.

IGF-1R is a receptor tyrosine kinase (RTK) that is widely expressed in human cells and tissues and regulates cell growth, proliferation and migration [10]. The mature IGF-1R is a heterotetramer consisting of two extracellular α subunits (IGF-1Rα) and two transmembrane β subunits (IGF-1Rβ), both encoded by a single gene on chromosome 15q26.3 [11]. The ligands of IGF-1R include IGF-1, IGF-2 and insulin [12], when IGF binds to the α subunit of IGF-1R, the conformation of the β subunit changes, which in turn triggers the autophosphorylation of its tyrosine kinase structural domain, and autophosphorylation further activates the kinase activity of IGF-1R [13]. In addition, other kinases such as FAK and Src can also phosphorylate and activate IGF-1R [14, 15]. The phosphorylation of IGF-1R can initiate signaling pathways including PI3K, MAPK and STAT3 [16–18], which regulate cellular physiological and pathological activities. It has been demonstrated that IGF-1R is abnormally elevated in approximately 50% of breast cancers and negatively correlates with patient prognosis [19, 20]. In addition, IGF-1R is overexpressed in approximately 30% of prostate cancers and is associated with an increased risk of tumor recurrence and metastasis [21]. In the in vitro experiments, inhibition of IGF-1R in nasopharyngeal carcinoma cells inhibited cell proliferation and increased the radiosensitivity of nasopharyngeal carcinoma cells [22]. However, the exact mechanism of IGF-1R in distant metastasis of nasopharyngeal carcinoma and its therapeutic implications are unknown.

Insulin-like growth factor (IGF), structurally similar to insulin, is an active protein peptide with multiple functions such as inducing cell growth, promoting protein synthesis, and maintaining cellular physiological activities. IGFs include IGF-1 and IGF-2, and previous studies have confirmed that IGFs derived from liver and adipose tissue acts in an endocrine manner while in other tissues in a paracrine or autocrine manner [11, 23]. IGF-1 can promote colorectal cancer metastasis by mediating HOXA13 overexpression [24], and IGF-1 promotes gastric cancer growth and metastasis by inducing IFITM2 [25]. Recently, it has been reported that IGF-1 secreted by osteoclasts promotes osteoblast differentiation and plays an important role in bone formation [26, 27]. However, the effects that IGFs play on other cells, especially tumor cells, in the bone are unclear, therefore further studies on the role played by IGFs in tumor bone metastasis are necessary.

Akt is essentially a serine/threonine kinase, also known as protein kinase B (PKB), and its dysregulation plays an important role in a variety of cancers, with increased Akt kinase activity detectable in gastric, prostate and breast cancers [28]. Various growth factors such as epidermal growth factor (EGF), insulin-like growth factor (IGF) and vascular endothelial growth factor (VEGF) can induce kinase activity of Akt [29]. When phosphorylated, Akt can initiate a wide range of downstream effectors, such as core proteins that regulate apoptosis, transcription factors, and oncogenic factors, including the mammalian target of rapamycin (mTOR). mTOR activation can target many molecules, including translation initiation factors, hypoxia-associated factors, factors associated with angiogenesis and factors that regulate the cell cycle [30]. The PI3K/Akt/mTOR signaling pathway has been proven to be activated in a variety of cancers [31], however its effect on the development of distant metastasis in nasopharyngeal carcinoma cells is unclear.

In our study, we demonstrated that nasopharyngeal carcinoma tumor cells with high IGF1-R expression have a high propensity for bone metastasis, and IGF-1 secreted by osteoclasts promotes the proliferation of tumor cells in the bone marrow through the IGF-1R/AKT/S6 signaling pathway. The GM-CSF secretion by tumor cells promotes osteoclast differentiation, leading to increased bone resorption.

## Materials and methods

### Enzyme-linked immunosorbent assay

Culture supernatants were collected from each group of cells. After removal of impurities, the supernatants were centrifuged at 1000 g for 20 min at 4°C. The processed supernatants were immediately used to measure the levels of IGF-1, IGF-2 and GM-CSF using ELISA kits.

### Quantitative real-time PCR analysis

Total RNA from tissues on paraffin sections was extracted using the RNAprep Pure FFPE Kit (TIANGEN) according to the manufacturer's instructions for subsequent experiments. Total RNA from cells was extracted using TRIzol reagent (TaKaRa, Japan), total RNA from tissues in paraffin sections was extracted using the kit according to the manufacturer's instructions, and cDNA was synthesized using the PrimeScript RT kit (TaKaRa). qRT-PCR was performed in triplicate using SYBR Premix ExTaq (TaKaRa). GAPDH were used to normalize mRNA expression. The 2-ΔΔCt method was used. Independent experiments were performed in triplicate and the primer sequences are listed in Supplementary Table 1.

### Western blotting analysis

The bone tissues were snap-frozen in liquid nitrogen and then crushed. The crushed bone tissues and cells were lysed in buffer containing protease inhibitors. The lysates were then subjected to sodium dodecyl sulfate polyacrylamide gel electrophoresis and transferred to polyvinylidene difluoride membranes. The membranes were blocked with 5% non-fat milk diluted in Tris-buffered saline for 2 h at room temperature. Next, the membranes were incubated with primary antibodies at 4°C overnight. After several washes, the membranes were incubated with secondary antibodies for 1.5 h at room temperature. The signal was detected using an enhanced chemiluminescence kit (Beyotime, Shanghai, China).

### Histopathological and immunostaining analysis (IHC)

Hematoxylin and eosin (H&E) staining was performed to evaluate the tumor area in the bone metastatic lesions. IHC was conducted on the paraffin-embedded sections of clinical NPC and xenograft mice tissues. The specimens were initially deparaffinized in xylene and rehydrated by an ethanol gradient. After incubation of sections with primary antibodies, expression levels were evaluated using the GTVisionTM III Detection System (Gene Tech) according to the manufacturer's instructions, and the staining intensity and extent were assessed using previously reported techniques [32].

### TRAP staining

TRAP staining of tissue sections and cells were performed using the TRAP kit (Wako), and multinucleated TRAP-positive cells were quantified.

### Cell experiments

EBV-negative NPC cell line 5–8F was generously provided by Prof. Musheng Zeng (Sun Yat-sen University Cancer Center, China). Primary mouse bone marrow cells were obtained from 4-week-old BALB/c nude mice. After culturing them for 6–8 days using α-MEM (containing 20% FBS, 50 ng/mL RANKL), mature osteoclasts were washed with PBS and then cultured with RANKL-free α-MEM (containing 1% FBS) for 24 h. The filtered supernatant was then collected and used as conditioned medium to culture tumor cells. IGF-1 neutralizing antibody (1μg/mL), recombinant IGF-1 antibody (100 ng/mL), NVP-AEW541 (10μM) or rapamycin (5nM) was added to some of the conditioned medium to treat the tumor cells for 48 h.

α-MEM (containing 20% FBS, 25 ng/mL RANKL) was mixed with CM from cancer cells in a 3:1 ratio for osteoclast differentiation. After culturing primary mouse bone marrow cells for 6 to 8 days, staining was performed using the TRAP kit. Cell viability was determined using the Cell Counting Kit-8 (CCK-8) assay.

Lentiviral particles carrying the IGF-1R vector (Ubi-MCS-3FLAG-CBh-gcGFP-IRES-puromycin-IGF-1R) and control vectors were constructed by GeneChem (Shanghai, China). The lentiviral transductions were carried out following the manufacturer’s protocol. For IGF-1R and CSF2 knockdown, the annealed sense and antisense shRNA oligonucleotides were cloned into the pLKO.1-puro vector (Addgene) with the following target sequences: CCGGGAGACAGAGTACCCTTTCTTTCTCGAGAAAGAAAGGGTACTCTGTCTCTTTTTG (shIGF-1R) and CCCAGATTATCACCTTTGAAA (shCSF2) [33, 34].

### Mouse experiments

The animal procedures in this study were approved by the Ethical Committee for Animal Research of Southern Medical University (Guangzhou, China).The BALB/c nude mice (3–4 weeks old, male) were purchased from the Central Animal Facility of the Southern Medical University. 8 × 10^5^ cells were injected into the left ventricle of the heart. Treatments for intracardiac injected mice were all started after two weeks and maintained for two weeks. The treatments were as follows: 1. Each mouse was injected with IGF1-neutralizing antibody (50 µg/mL in PBS, 5 µL/mouse) every other day. 2. Each mouse was gavaged twice daily with NVP-AEW541 (20 mg/kg). 3. Each mouse was injected intraperitoneally with rapamycin (5 mg/kg) daily. BLI data were acquired by Ami HTX (Spectral Instruments Imaging). Micro-CT data acquired by uCT80 (Bruker) system. RNA sequencing was performed to identify differentially expressed genes between 5-8F and BM3 cells. Bone tissue was collected for paraffin embedding and sectioning. HE staining was used to assess tissue condition. TRAP staining was performed with the TRAP kit to assess the number of osteoclasts.

### Clinical analysis

Samples of the nasopharyngeal carcinoma cohort were obtained from the Nanfang Hospital of Southern Medical University, and bone metastases were diagnosed by imaging. RNA was extracted from paraffin-embedded tissues for IGF-1R expression analysis. Immunostaining for IGF-1R, IGF-1 and pS6 was performed. Baseline characteristics, inclusion and exclusion criteria of the nasopharyngeal carcinoma sample cohort and study cohort were described in detail in Supplementary Table 2 and 3. Patients have given their informed consent, *Declaration of Helsinsky* has been followed in this study, and all procedures in this study were approved by the Ethics Committee of the Nanfang Hospital (Guangzhou, China).

The antibodies and reagents in this study are listed in Supplementary Table 4.

### Statistical analysis

Data were analyzed using SPSS 19.0 and GraphPad Prism 8.0 (GraphPad Software Inc.), Results are expressed as mean ± SD of all measured parameters. *P* < 0.05 was considered statistically significant. Comparisons between two groups were performed using Student's t-test, while one-way ANOVA was used for multiple group comparisons. The Kaplan–Meier method and one-way analysis of variance were used to estimate survival curves. Single, double, triple and quadruple asterisks indicate statistical significance.

## Results

### 1 IGF-1R expression is associated with NPC bone metastasis

**Fig. 1:**
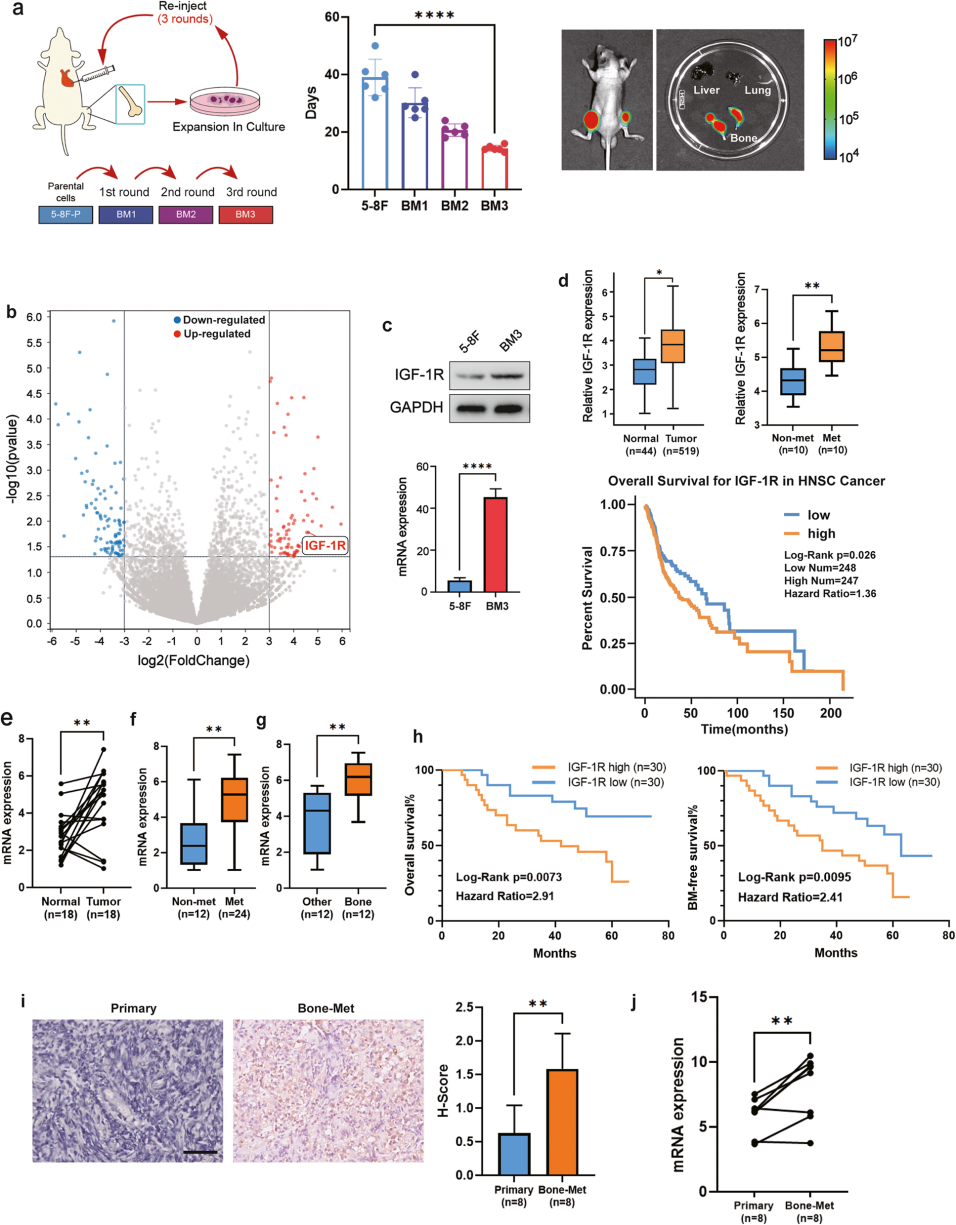
IGF-1R expression is associated with bone metastasis of NPC. **a** Pattern plot of BM3 cells obtained, time of formation of bone metastatic lesions in each cell lines, representative BLIs of BM3 bone metastasis formation. **b** Volcano plot showing significantly altered genes (upregulation or downregulation with fold-change > 8, *p* < 0.05) in BM3 compared to 5-8F. **c** IGF-1R protein and mRNA levels in 5-8F and BM3 cell lines. **d** Expression and survival analysis of IGF-1R in a cohort study of head and neck squamous cell (HNSC) cancer patients in the TCGA database. IGF-1R expression in patients with and without distant metastases in the GSE180272 dataset. **e** IGF-1R expression in paired normal and cancer tissues from the same patient. **f** IGF-1R expression in metastatic and non-metastatic primary NPC tumors. **g** IGF-1R expression in primary NPC tumors metastatic to bone or other organs. **h** Overall survival and bone metastasis–free survival analyses of the patients according to different IGF-1R expression status. **i** Representative images and quantitation of IGF-1R immunostaining in primary NPC tumors and bone metastases from the same patient. **j** IGF-1R mRNA levels in primary NPC tumors and bone metastases from the same patients. (“ns” indicates *P* > 0.05, “*” represents *P* < 0.05, “**” represents *P* < 0.01,“***” represents *P* < 0.001 and “****” represents *P* < 0.0001, Scar bar = 50 μm.)

To study the bone metastasis of nasopharyngeal carcinoma, we established a high bone metastasis-prone 5-8F-derived cell line [35]. By injecting parental 5-8F cells into the left ventricle of mice, waiting until the mice developed bone metastasis, collecting nasopharyngeal carcinoma cells at the site of bone metastasis for expansion and culture, and performing the next round of intracardiac injection, we finally obtained a high bone metastasis-prone 5-8F-derived cell line named BM3, the establishment process of BM3 cell line was described in detail in Supplementary Materials and Methods. BM3 showed strong metastatic signal only in the bones of mice, but not in other organs. BM3 induced bone metastasis and osteolysis more rapidly after injection into the left ventricle of mice (Fig. 1a). Next, we performed RNA-Seq analysis of the parental 5-8F cell line and BM3. To explore the tumor-microenvironment interactions, we paid particular attention to the genes encoding cell membrane proteins (The screening process is described in Supplementary Table 5, Supplementary Fig. 1a, b). After screening, we found that IGF-1R was significantly upregulated in BM3 compared to parental 5-8F (Fig. 1b). Upregulation of IGF-1R in BM3 was confirmed at the RNA and protein levels (Fig. 1c). We then assessed the clinical relevance of differentially expressed genes by analyzing the TCGA dataset. IGF-1R expression was significantly upregulated in HNSCC tissues compared to normal tissues (http://gepia.cancer-pku.cn/index.html) and was associated with shorter patient survival (https://starbase.sysu.edu.cn/index.php). In addition, we analyzed the published GSE180272 dataset [36] and found that IGF-1R was significantly upregulated in tissues from patients who had developed distant metastases compared to those who had not (Fig. 1d). We then further validated the clinical relevance of IGF-1R in a cohort of nasopharyngeal carcinoma at Nanfang Hospital of Southern Medical University. The analysis showed that IGF-1R expression was significantly higher in tumor tissue compared with paracancerous tissue from the same patient (Fig. 1e, Supplementary Fig. 2). IGF-1R was also more upregulated in metastatic tumors than in non-metastatic tumors (Fig. 1f). In metastatic tumors, bone metastasis-prone IGF-1R levels were much higher than those of tumors prone to metastasis to other organs (Fig. 1g). In addition, patients with higher IGF-1R expression had a higher risk of bone recurrence and poorer overall survival (Fig. 1h). In addition, IGF-1R expression was higher in patients with bone metastases from nasopharyngeal carcinoma than in primary tumors (Fig. 1i). This was also verified in the same patient (Fig. 1j). These findings reveal association between IGF-1R and bone metastasis in nasopharyngeal carcinoma.

### 2 IGF-1R promotes bone metastasis of NPC

**Fig. 2:**
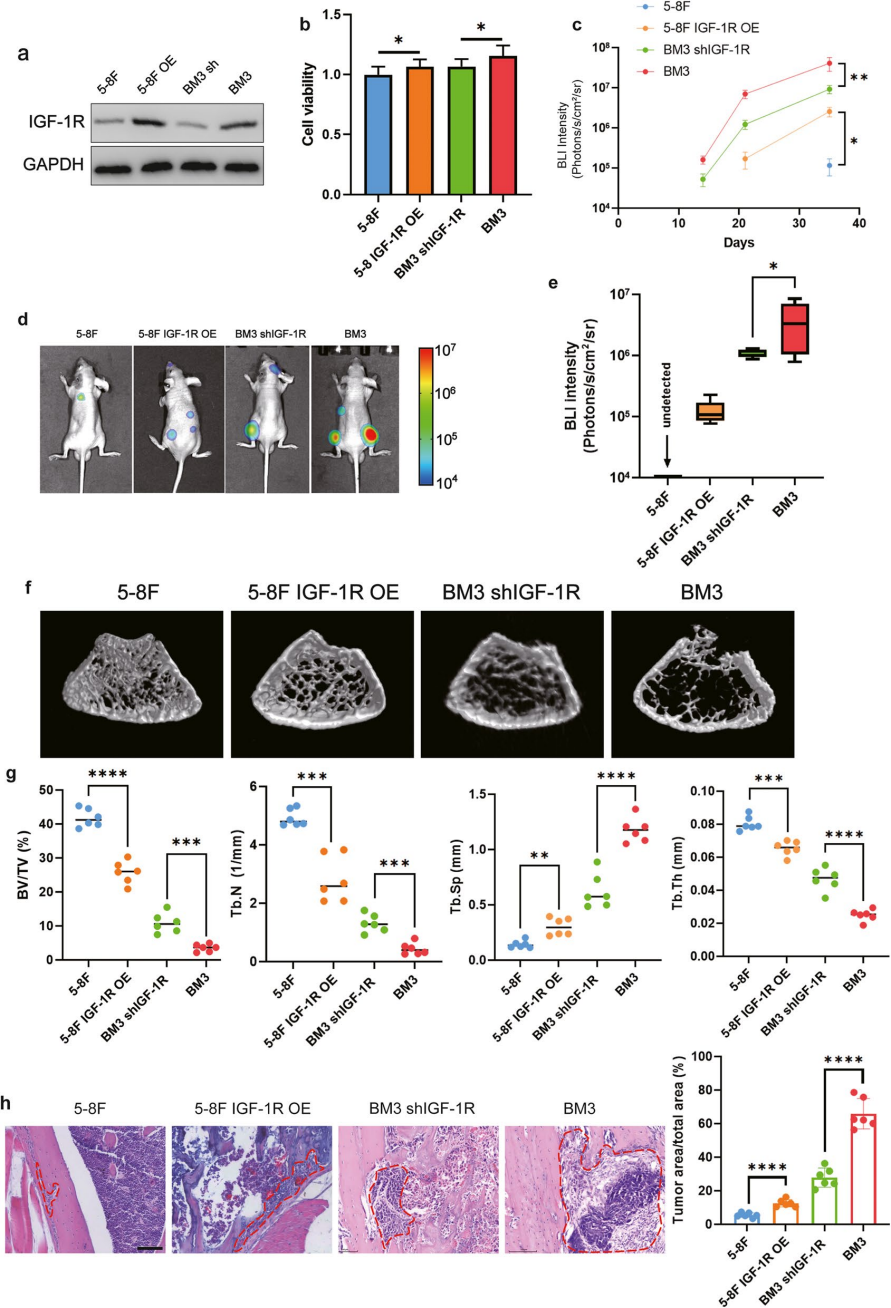
IGF-1R promotes bone metastasis of NPC. **a** IGF-1R overexpression in 5-8F and knockdown in BM3. **b** CCK8 assay of cell lines to evaluate cell viability. **c** BLI quantitation of tumor burden of the mice. **d** Representative images of BLI of whole bodies. **e** BLI analysis of femurs. **f** Representative micro-CT images of femurs. **g** Micro-CT analyses for bone destruction of femurs. **h** H&E representative images and quantitative analysis of bone metastasis. (Tumor tissue is noted in the red line, “ns” indicates *P* > 0.05, “*” represents *P* < 0.05, “**” represents *P *< 0.01,“***” represents *P* < 0.001 and “****” represents *P* < 0.0001, Scar bar = 100 μm.)

To explore the functional role of IGF-1R in bone metastasis, IGF-1R was stably overexpressed in 5-8F and knocked down in BM3 (Fig. 2a). We first found that overexpression of IGF-1R in 5-8F resulted in enhanced cell proliferation compared to 5-8F, and knockdown of IGF-1R in BM3 inhibited its proliferation (Fig. 2b). When these cells were intracardiacally inoculated into immunodeficient nude mice, IGF-1R overexpression in 5-8F significantly accelerated bone metastasis, leading to increased bone destruction. IGF-1R knockdown in BM3 cells reduced the rate of bone metastasis and bone destruction (Fig. 2c-h). These findings reveal a bone metastatic role for IGF-1R in nasopharyngeal carcinoma.

### 3 IGF-1 secreted by osteoclasts promotes the proliferation of IGF-1R-expressing nasopharyngeal carcinoma cells

**Fig. 3:**
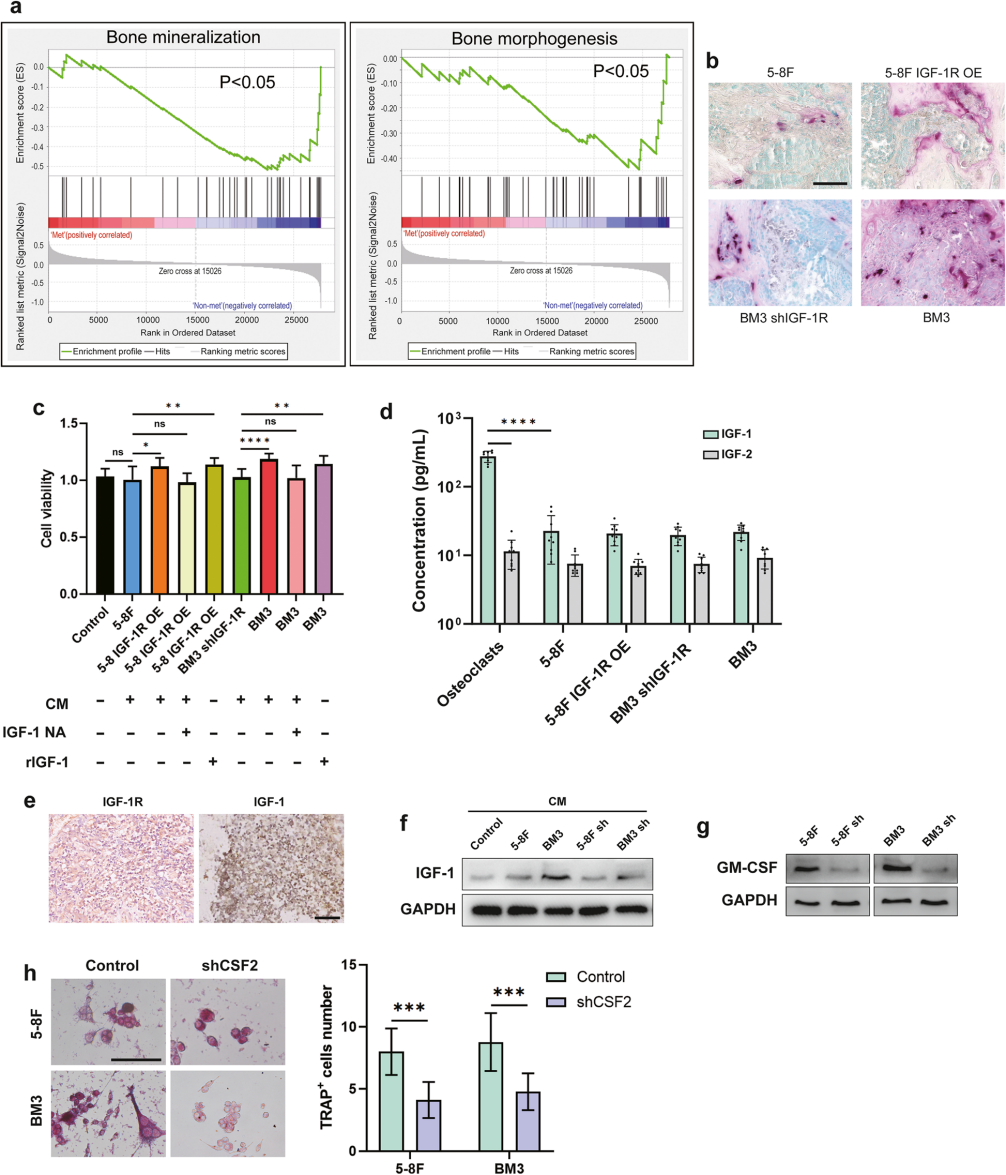
IGF-1 secreted by osteoclasts promotes the proliferation of IGF-1R-expressing nasopharyngeal carcinoma cells. **a** GSEA of the “Bone mineralization” and “Bone morphogenesis” gene modules in NPC patients of GSE180272 with metastasis or non-metastasis. **b** Representative TRAP staining of osteoclasts along the tumor-bone interface of bone metastases. **c** CCK8 assay of cell lines with/without treatment of CM from murine bone marrow–derived osteoclasts (OC) for 48 h, with/without IGF-1 neutralizing antibody (1 μg/mL) or rIGF-1(100 ng/mL). **d** Secretion level of IGF-1 and IGF-2 in osteoclasts and tumor cells. **e** IGF-1R and IGF-1 immunostaining of bone metastases from NPC patients. **f** IGF-1 secretion of osteoclasts after treatment with control normal medium or CM from 5-8F or BM3 with or without CSF2 knockdown. **g** GM-CSF secretion in 5-8F and BM3 cells with/without CSF2 knockdown. **h** Osteoclastogenesis assays from murine bone marrow when the bone marrow was treated with CM from 5-8F or BM3 with or without CSF2 knockdown. (“ns” indicates *P* > 0.05, “*” represents *P* < 0.05, “**” represents *P* < 0.01,“***” represents *P* < 0.001 and “****” represents *P *< 0.0001, Scar bar = 50 μm.)

Next, we found that the occurrence of distant metastases in nasopharyngeal carcinoma was mainly negatively correlated with bone mineralization and bone morphogenesis (Fig. 3a) by GSEA analysis of the GSE180272 dataset (the related genes are listed in Supplementary Table 6). The occurrence of bone metastasis in nasopharyngeal carcinoma often induced the occurrence of osteolysis in clinical practice, and we examined the presence of mature osteoclasts at the lesion of bone metastatic carcinoma in animal models by tartrate-resistant acid phosphatase staining (TRAP) (Fig. 3b). When tumor cells were treated with conditioned medium (CM) of osteoclasts, the proliferation of 5-8F OE cells was significantly stimulated by osteoclastic CM, as measured by CCK8, whereas control 5-8F demonstrated no response. Similarly, BM3 responded significantly to CM stimulation, and the response was reduced after IGF-1R knockdown (Fig. 3c). Next, we tried to find out which factor in osteoclastic CM triggers the proliferative response of cancer cells. The ligands of IGF-1R include IGF-1 and IGF-2 [37]. We found that only IGF-1 was abundantly expressed by osteoclasts, while IGF-2 was barely expressed. In addition, tumor cells expressed much lower levels of IGF-1 than osteoclasts (Fig. 3d). Importantly, immunostaining of bone metastases from clinical nasopharyngeal carcinoma showed that IGF-1R was abundantly expressed in tumor cells and IGF-1 in microenvironmental cells (Fig. 3e). These data suggest that osteoclast-derived IGF-1 may be a key factor in the stimulation of nasopharyngeal carcinoma cells. To confirm this hypothesis, the addition IGF-1 neutralizing antibody in osteoclastic CM blocked the enhanced proliferation of 5-8F OE and BM3. In addition, recombinant IGF-1 promoted the proliferation of 5-8F OE and BM3, while 5-8F and BM3sh demonstrated no response (Fig. 3c).

In addition, IGF-1 secretion by osteoclasts was enhanced by CM of 5-8F and BM3 when osteoclasts were cultured in tumor cell CM (Fig. 3f). Since osteoclasts were reported to secrete IGF-1 in the skeletal microenvironment, and GM-CSF has been reported to induce IGF-1 expression [38], and tumor cells usually produce GM-CSF abundantly, we explored whether IGF-1 expression of osteoclast is regulated by GM-CSF. Indeed, both 5-8F and BM3 cells secreted GM-CSF (Fig. 3g). In addition, when the GM-CSF gene CSF2 was knocked down in 5-8F and BM3 (Fig. 3g) to inhibit GM-CSF secretion, their CM-promoted osteoclast IGF-1 production was weakened (Fig. 3f). CSF2 knockdown also inhibited tumor-induced osteoclast maturation, confirming the role of GM-CSF in osteolysis (Fig. 3h). In summary, these data suggest that tumor cells can induce IGF-1 secretion by osteoclasts and that osteoclast-derived IGF-1 in turn promotes the proliferation of IGF-1R-expressing nasopharyngeal carcinoma cells.

### 4 IGF-1 activates AKT/S6 signaling in tumor cells, depletion of IGF-1 in mice inhibits bone metastasis

**Fig. 4:**
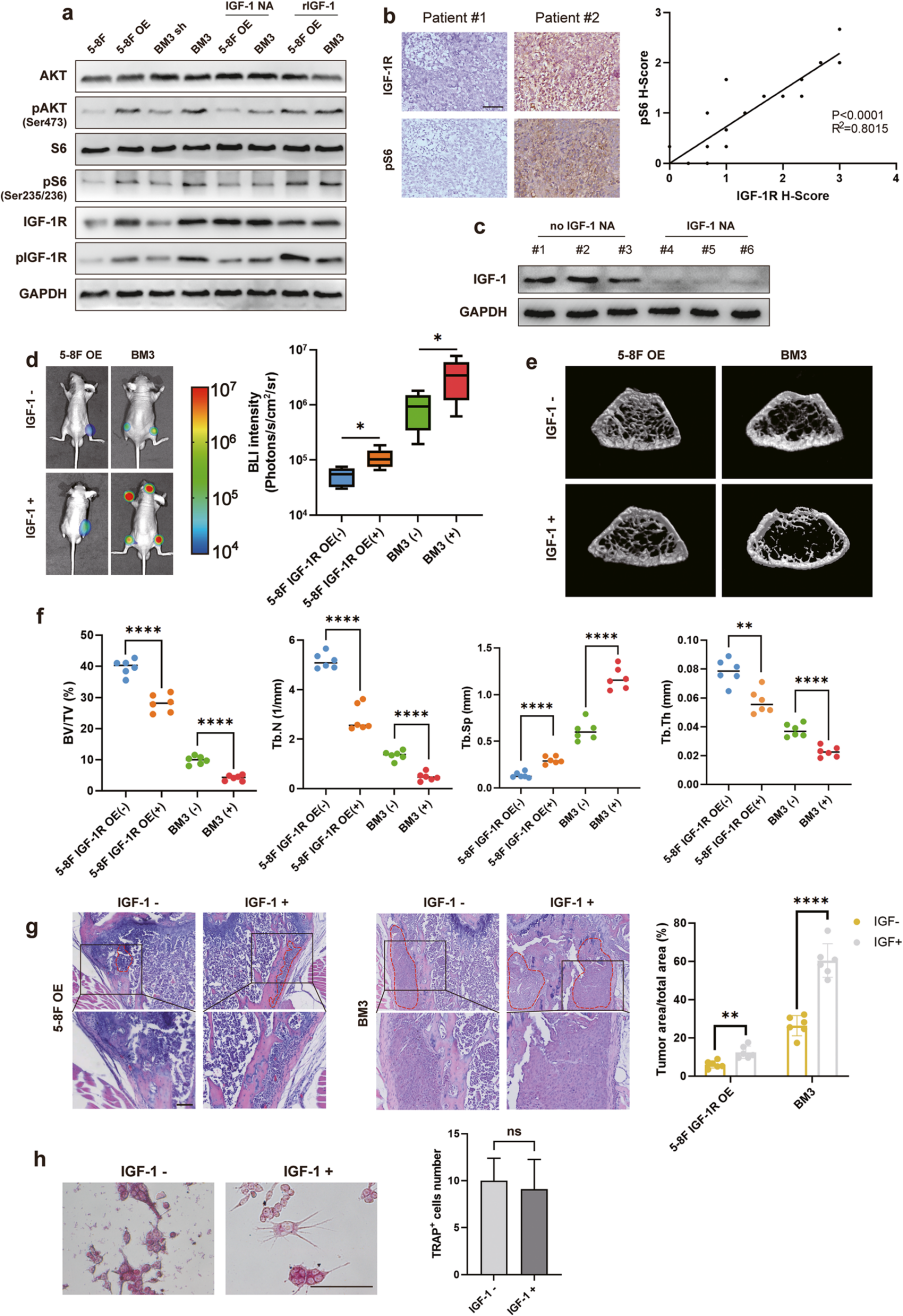
IGF-1 activates AKT/S6 signaling in tumor cells, depletion of IGF-1 in mice inhibits bone metastasis. **a** The protein levels of AKT, pAKT (Ser473), S6, pS6 (Ser235/236), IGF-1R and pIGF-1R in 5-8F, 5-8F OE, BM3 sh and BM3 cells cultured in osteoclastic CM with/without IGF-1 neutralizing antibody (IGF-1 NA) and/or recombinant IGF-1 (rIGF-1). **b** Representative immunostaining images of IGF-1R and S6 phosphorylation and correlation between IGF-1R expression and S6 phosphorylation in the NPC cohort. **c** IGF-1 expression in bone marrow with/without IGF-1 neutralizing antibody. **d** Intracardiac injection of 5-8F IGF-1R OE or BM3 into mice with/without IGF-1 being neutralized for bone metastasis analysis. Representative BLIs and analysis of bone metastases. **e** Representative micro-CT images of femurs. **f** Micro-CT analyses for bone destruction of femurs. **g** Representative H&E images and analysis of bone metastases. **h** Osteoclastogenesis of primary bone marrow cells in mice with/without IGF-1 being neutralized. (Tumor tissue is noted in the red line, “IGF-1 NA” represents IGF-1 neutralizing antibody, “rIGF-1” represents recombinant IGF-1, “IGF-1 -” represents IGF-1 was neutralized, "IGF-1 + " represents IGF-1 was not neutralized. “ns” indicates *P* > 0.05, “*” represents *P* < 0.05, “**” represents *P* < 0.01,“***” represents *P* < 0.001 and “****” represents *P* < 0.0001, Scar bar = 50 μm.)

IGF-1 has been demonstrated to activate the mTORC1 signaling pathway, which plays a crucial role in cancer cell proliferation and tumor progression. Therefore, we explored whether osteoclast-derived IGF-1 activates the mTORC1 pathway in nasopharyngeal carcinoma cells. Using osteoclastic CM treated four cells, phosphorylation of AKT and S6 was elevated in 5-8F OE, but not in control 5-8F. Similarly, phosphorylation of AKT and S6 was elevated in BM3 compared to BM3 sh. IGF-1 neutralizing antibody effectively blocked osteoclast-induced phosphorylation of AKT and S6 in 5-8F OE and BM3 cells, while recombinant IGF-1 restored phosphorylation of AKT and S6 in both (Fig. 4a). Notably, the site where AKT undergoes phosphorylation is Ser473 rather than Thr308, and the site where S6 undergoes phosphorylation is Ser235/236 rather than Ser240/244 (Fig. 4a, Supplementary Fig. 3). In addition, immunostaining of clinical nasopharyngeal carcinoma samples from the Nanfang Hospital of Southern Medical University cohort revealed a strong positive correlation between IGF-1R expression and phosphorylation of S6 (Fig. 4b). These data suggest that the mTORC1 signaling pathway is activated by IGF-1 secreted by osteoclasts in IGF-1R-expressing nasopharyngeal carcinoma cells.

To further validate the function of the IGF-1/IGF-1R signaling in bone metastasis, 5-8F cells overexpressing IGF-1R and BM3 were intracardiacally inoculated into immunodeficient nude mice, and in a subset of these mice, injected with IGF-1 neutralizing antibody [39]. Reduced expression of IGF-1 in the bone marrow of mice injected with IGF-1 neutralizing antibody was confirmed by protein blotting (Fig. 4c). In the presence of IGF-1 being neutralized, bone metastasis was inhibited in BM3 cells and 5–8 cells overexpressing IGF-1R, and the bone destruction was significantly reduced (Fig. 4d-g). Furthermore, when primary bone marrow cells were isolated from mice with or without IGF-1 neutralizing antibody injection, they showed no obvious difference in differentiation into mature osteoclasts (Fig. 4h). However, osteoclastic CM supplemented with IGF-1 neutralizing antibody lost the ability to promote proliferation of 5-8F cells overexpressing IGF-1R and BM3 cells (Fig. 3c). These data suggest that microenvironmentally-derived IGF-1 is essential for bone metastasis in nasopharyngeal carcinoma.

### 5 The mTORC1 inhibition suppresses IGF-1R–induced tumor proliferation and bone metastasis

**Fig. 5:**
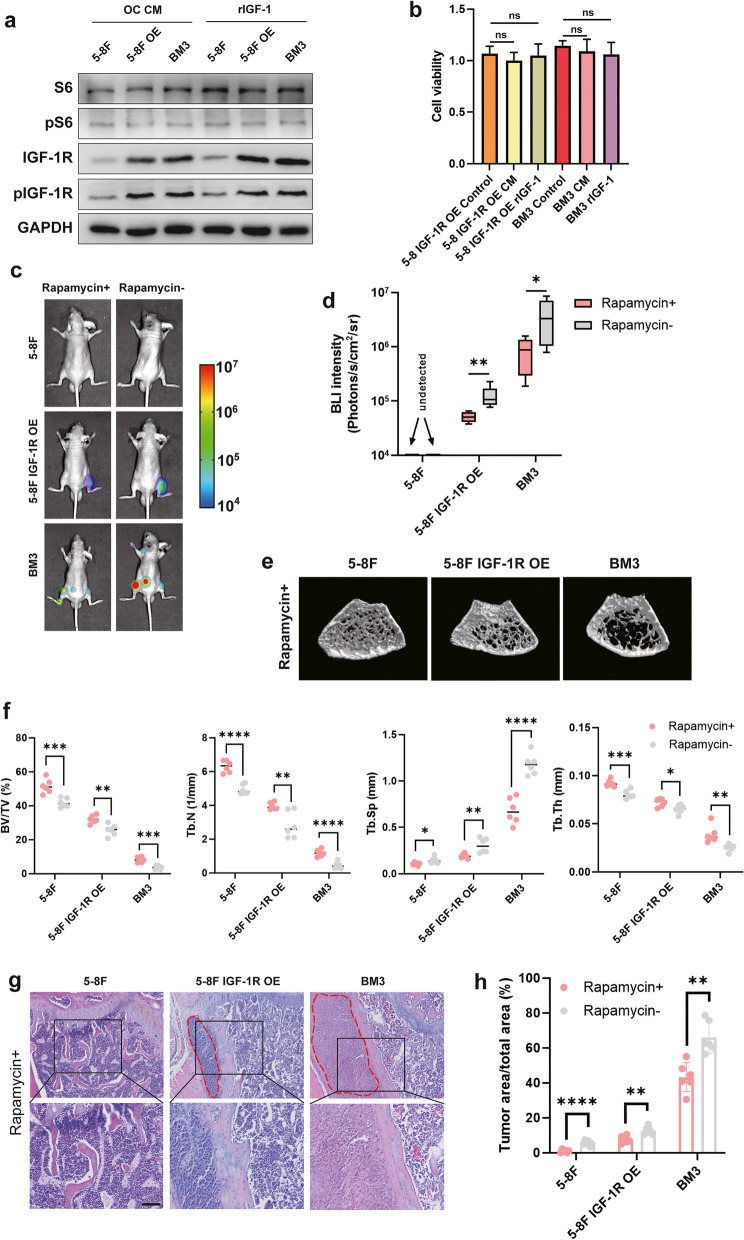
The mTORC1 inhibition suppresses IGF-1R–induced tumor proliferation and bone metastasis. **a** The protein levels of S6, pS6, IGF-1R and pIGF-1R in 5-8F, 5-8F OE and BM3 cells cultured in osteoclastic CM or routine medium with recombinant IGF-1 after rapamycin treatment. **b** CCK8 assay of cell lines cultured in osteoclastic CM or routine medium with recombinant IGF-1 after rapamycin treatment. **c** Intracardiac injection of 5-8F,5-8F IGF-1R OE or BM3 into mice with/without rapamycin treatment for bone metastasis analysis. Representative BLIs of bone metastases. **d** BLIs analysis of bone metastases. **e** Representative micro-CT images of femurs. **f** Micro-CT analyses for bone destruction of femurs. **g** Representative H&E images of bone metastases. **h** H&E analysis of bone metastases. (Tumor tissue is noted in the red line, “ns” indicates *P* > 0.05, “*” represents *P* < 0.05, “**” represents *P* < 0.01,“***” represents *P* < 0.001 and “****” represents *P* < 0.0001, Scar bar = 50 μm.)

To further define whether mTORC1 acts downstream of IGF-1R in regulating bone metastasis, mTORC1 specific inhibitor rapamycin [40] was added to osteoclastic CM or routine medium with recombinant IGF-1. Rapamycin eliminated osteoclastic CM or recombinant IGF-1 induced phosphorylation of S6 in 5-8F, 5-8F OE and BM3 tumor cells (Fig. 5a), and when mTORC1 was inhibited, osteoclastic CM or recombinant IGF-1 failed to promote proliferation of 5-8F OE, and BM3 tumor cells (Fig. 5b). When these three cells were intracardiacally inoculated into immunodeficient nude mice, rapamycin treatment attenuated the rate of bone metastasis, bone metastatic tumor burden, and bone destruction (Fig. 5c-h). Overall, these data suggest that blocking mTORC1 effectively inhibits IGF-1R-induced bone tumor proliferation and reduces the risk of bone metastasis.

### 6 IGF-1R inhibitor effectively inhibits bone metastasis in nasopharyngeal carcinoma

**Fig. 6:**
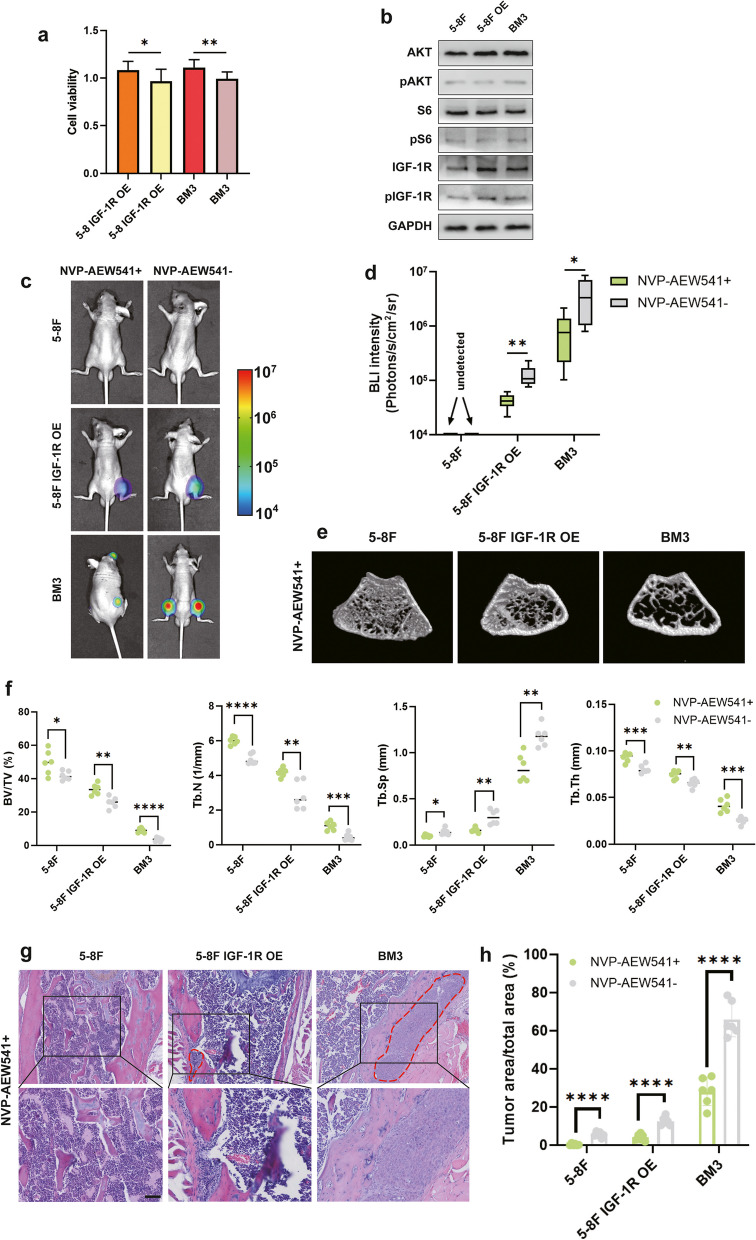
IGF-1R inhibitor effectively inhibits bone metastasis in nasopharyngeal carcinoma. **a** CCK8 assay of 5-8F OE and BM3 cell lines cultured in osteoclastic CM with or without NVP-AEW541. **b** The protein levels of AKT, pAKT, S6, pS6, IGF-1R and pIGF-1R in 5-8F, 5-8F OE and BM3 cells cultured in osteoclastic CM with NVP-AEW541. **c** Intracardiac injection of 5-8F,5-8F IGF-1R OE or BM3 into mice with/without NVP-AEW541 for bone metastasis analysis. Representative BLIs of bone metastases. **d** BLIs analysis of bone metastases. **e** Representative micro-CT images of femurs. **f** Micro-CT analyses for bone destruction of femurs. **g** Representative H&E images of bone metastases. **h** H&E analysis of bone metastases. (Tumor tissue is noted in the red line, “ns” indicates *P* > 0.05, “*” represents *P* < 0.05, “**” represents *P* < 0.01,“***” represents *P* < 0.001 and “****” represents *P* < 0.0001, Scar bar = 50 μm.)

The specific high expression of IGF-1R in nasopharyngeal carcinoma tumor and low expression in normal tissue suggest the potential of IGF-1R as a therapeutic target. Addition of IGF-1R inhibitor NVP-AEW541 [41, 42] to osteoclast CM, used to culture 5-8F OE and BM3, effectively inhibited cell proliferation (Fig. 6a), and suppressed phosphorylation of IGF-1R, AKT and S6 (Fig. 6b). We then tested the efficacy of IGF-1R inhibitors in the treatment of bone metastasis in vivo. 5-8F, 5-8F OE and BM3 cells were intracardiacally inoculated into immunodeficient nude mice, treatment with the NVP-AEW541 resulted in a substantial reduction in tumor bone metastases, a significant reduction in tumor load, and diminished bone destruction (Fig. 6c-h). These data collectively confirm the efficacy of the inhibitor NVP-AEW541 for the treatment of NPC bone metastasis.

## Discussion

Circulating tumor cells are present in the bone marrow in an active or dormant state long before the onset of clinically detectable metastasis. Tumor cells interact with various cell populations in the bone marrow such as vascular endothelial cells, osteoclasts, osteoblasts, immune cells and adipocytes to modify the bone microenvironment and prepare for further tumor development [43]. Bone metastasis is one of the most common complications of early-stage nasopharyngeal carcinoma, often resulting in adverse consequences such as fractures and bone pain, which seriously affect patients' quality of life and cause great psychological burden. Therefore, the cellular and molecular mechanisms underlying the predisposition of nasopharyngeal carcinoma to bone metastasis at an early stage urgently need further study. Due to the lack of animal models of nasopharyngeal carcinoma bone metastasis, we established an experimental bone metastasis model by injecting a 5-8F cell line into the left ventricle of nude mice, and obtained a cell line with high propensity for bone metastasis by multiple rounds of intracardiac injection. Although previous studies have reported the biological function of IGF-1R in many cancers, including nasopharyngeal carcinoma, its potential mechanism in NPC bone metastasis has not been studied. Cells expressing prometastatic gene features are present early in tumorigenesis [44]. In our study, elevated expression of IGF-1R was highly correlated with the development of bone metastasis in NPC patients and resulted in a worse survival prognosis. In animal experiments, cells with high IGF-1R expression were predisposed to bone metastasis, had a shorter time to develop bone metastasis, and resulted in more severe bone destruction. These results suggest that IGF-1R may be a biomarker for predicting the occurrence of bone metastasis in nasopharyngeal carcinoma in situ. Targeting of IGF-1R in animal models can effectively inhibit/delay the development of bone metastasis. In addition, according to the "soil theory", before circulating tumor cells reach the bone, the bone microenvironment has been altered to become a "soil" suitable for colonization by circulating tumor cells [43]. We found for the first time that one of the ligands binding to IGF-1R of NPC cells in the bone microenvironment was IGF-1 secreted by osteoclasts rather than IGF-2. Whether there are other sources of IGF-1 remains to be further explored. Neutralizing antibody therapy targeting IGF-1 can reduce bone metastasis and osteolysis. NPC patients often already have bone metastasis at the time of initial diagnosis, which is of interest to many researchers. We found that the secretion of IGF-1 by osteoclasts promoted the proliferation of NPC cells, while the secretion of GM-CSF by NPC cells promoted the maturation and differentiation of osteoclasts, that is to say, IGF-1R mediated the crosstalk between the two types of cells, forming a malignant cycle that resulted in the rapid progression of bone metastasis and the further aggravation of bone destruction. When IGF-1R is activated, which means phosphorylation occurs, it affects tumor physiological activities through multiple downstream pathways, in this study, IGF-1R regulates NPC cell proliferation in bone through the AKT/mTORC1/S6 signaling axis, it is worth noting that the site where AKT undergoes phosphorylation is Ser473, rather than Thr308, and the site where S6 undergoes phosphorylation is Ser235/236, rather than Ser240/244. When this pathway was blocked with rapamycin, bone metastasis and bone destruction were reduced. Further preclinical and clinical studies are needed to validate the safety and efficacy of targeting IGF-1R and other components of the signaling axis for the treatment of bone metastasis. Overexpression and activation of IGF-1R has been reported to be associated with a high risk of metastasis and poor prognosis in many cancer patients [45]. IGF-1R, a receptor tyrosine kinase, promotes cancer development and metastasis by mediating ligand-independent activation of MET (receptor tyrosine kinase) in prostate cancer [46]. In pancreatic cancer, IGF-1R activates RON to promote cancer metastasis [47]. In breast cancer, IGF-1R drives the occurrence of EMT (epithelial-mesenchymal transition) through FAK and NF-kappaB, which in turn helps cancer cells to invade and circulate for survival [48, 49]. In addition, when tumor cells are separated from the stroma, anoikis may occur in the process of metastasis of circulating tumor cells, and anoikis can inhibit the metastasis of tumor cells. It has been demonstrated that inhibition of IGF-1R enhances the occurrence of anoikis in tumor cells and reduces the number of tumor cells in the blood circulation of mice, thereby inhibiting tumor metastasis [50]. In human breast cancer cells, upregulation of IGF-1R expression was reported to resist anoikis by inhibiting activation of p53 and p21 [51], and IGF-1R/Akt signaling promoted LIP expression to inhibit anoikis [52], and IGF-1R mediated Akt activation through RACK1 to promote anchor growth of breast cancer cells [53]. It has been shown that IGF-1R upregulation can lead to resistance to radiation therapy, and the mechanism is that IGF-1R promotes the repair of radiation-induced DNA double-strand breaks through its downstream molecules Akt and mTOR [18, 54]. It is well known that nasopharyngeal carcinoma has a high sensitivity to radiation therapy, however, whether nasopharyngeal carcinoma cells with high IGF-1R expression have radiotherapy resistance is not yet clear. Notably, the phenomenon of "abscopal effect" in clinical practice, in which radiation at one site may lead to tumor regression at distant and non-irradiated sites [55], is of interest to many researchers. However, studies on the abscopal effect of nasopharyngeal carcinoma bone metastasis are not common due to the lack of stable experimental animal models. In this study, we established a stable animal model of nasopharyngeal carcinoma bone metastasis, based on which it can help to study the abscopal effect, which is one of our next research directions. In conclusion, the novelty of this study is that it demonstrates that the interaction between nasopharyngeal carcinoma cells and osteoclasts leads to the occurrence of bone metastasis and aggravates bone destruction, which form a vicious circle with serious consequences. This also provides a new theoretical support for the phenomenon that nasopharyngeal carcinoma patients develop bone metastasis at an early stage and have a poor prognosis after that occurrence, and also provides a new target for the clinical treatment of nasopharyngeal carcinoma bone metastasis. However, there are some limitations in this study. The mechanism of IGF-1R overexpression in nasopharyngeal carcinoma cells in situ before and after the development of bone metastasis needs to be further investigated. A full understanding of the upstream mechanism of IGF-1R can better explain the occurrence of bone metastasis. In addition, whether treatment targeting IGF-1R in nasopharyngeal carcinoma cells before bone metastasis occurs can effectively prevent its occurrence, which still needs further study. The modification of the microenvironment by tumor cells is extremely complex, while osteoclasts, osteoblasts, immune cells and adipocytes are present in the bone. The present study only investigated the interaction between tumor cells and osteoclasts, and whether the two are influenced by the regulation of other cells is also a subject of interest to many researchers.

## Conclusion

**Fig. 7:**
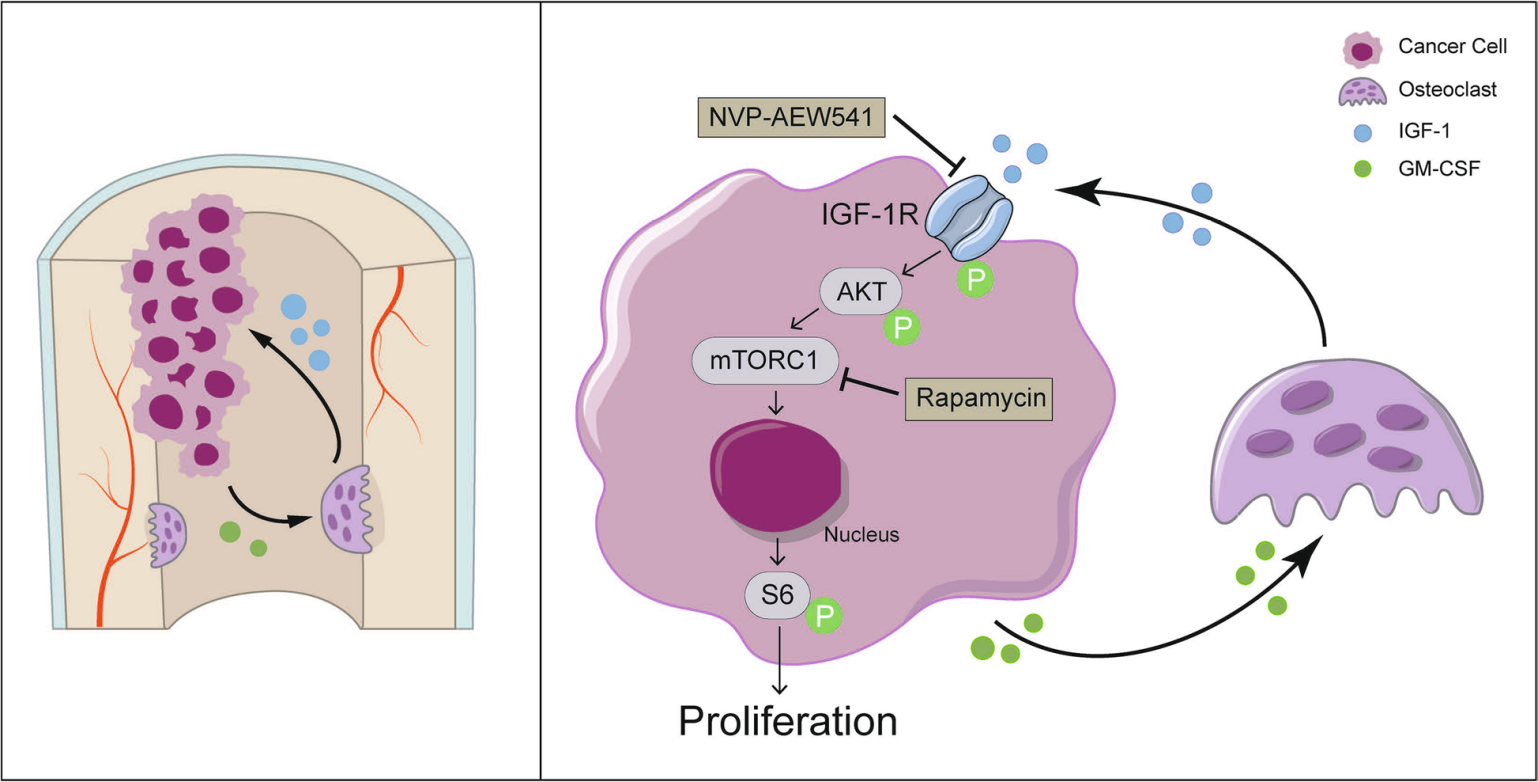
Potential mechanism by which IGF-1R mediates crosstalk between nasopharyngeal carcinoma cells and osteoclasts

In summary, our study reveals a novel interplay between nasopharyngeal carcinoma (NPC) cells and osteoclasts, demonstrating that this interaction promotes the occurrence of bone metastasis and exacerbates bone destruction, creating a vicious cycle with severe consequences. The elevated expression of IGF-1R in NPC cells is identified as a potential biomarker predicting bone metastasis in situ. Additionally, we elucidate that IGF-1 secreted by osteoclasts serves as a crucial component of the "soil" facilitating NPC cell colonization in bone, activating the IGF-1R/Akt/S6 signaling pathway to enhance proliferation. Furthermore, NPC cells secrete GM-CSF, promoting osteoclast differentiation and augmenting bone resorption, further contributing to bone destruction. Notably, specific inhibition of IGF-1R, mTORC, and neutralization of IGF-1 in animal models effectively disrupt the NPC cell-osteoclast interaction, delaying the onset of bone metastasis and reducing bone dissolution in metastatic lesions (Fig. 7).

### Supplementary Information

**Additional file 1: Supplementary Table 1.** Primer sets used for qPCR.** Supplementary Table 2.** Clinicopathological characteristics of the nasopharyngeal carcinoma sample cohort.** Supplementary Table 3.** Clinicopathological characteristics of the nasopharyngeal carcinoma study cohort.** Supplementary Table 4.** Antibodies and reagents used in this study.** Supplementary Table 5.** The expression of up-regulated genes located in the Plasma membrane were screened.** Supplementary Table 6.** Gene sets used for GSEA.** Supplementary Figure 1.** There was no significant difference in survival analysis and in the expression of normal versus tumor tissues of the candidate genes. (a) Survival analysis of candidate genes. (b) Expression of candidate genes in tumor tissues versus normal tissues. (“*” represents *P* < 0.05).** Supplementary Figure 2.** Quantitation of IGF-1R immunostaining in paired normal and cancer tissues from the same patient. (“***” represents *P* < 0.001).** Supplementary Figure 3.** The protein levels of AKT, pAKT (Thr308), S6 and pS6 (Ser240/244) in 5-8F and 5-8F OE cells cultured in osteoclastic CM. There was no significant difference in the protein levels of pAKT (Thr308) and pS6 (Ser240/244) between the two groups of cells. Supplementary Materials and Methods.

## Data Availability

The datasets used and/or analysed during the current study are available from the corresponding author on reasonable request.

## Abbreviations

IGF-1R: Insulin-like growth factor 1 receptor
NPC: Nasopharyngeal carcinoma
GM-CSF: Granulocyte–macrophage colony-stimulating factor
IGF-1R NA: IGF-1 neutralizing antibody
IGF-1: Insulin-like growth factor 1
OS: Overall survival
RTK: Receptor tyrosine kinase
AKT/Akt: Protein kinase B
mTOR: Mammalian target of rapamycin
ELISA: Enzyme-linked immunosorbent assay
OC: Osteoclast
CM: Conditioned medium
S6: Ribosomal protein S6
α-MEM: α-Minimum Essential Medium
BLI: Bioluminescence imaging
Tb.N: Trabecular number
Tb.Sp: Trabecular separation
Tb.Th: Trabecular thickness
BV/TV: Bone volume relative to total tissue volume
TRAP: Tartrate resistant acid phosphatase
pIGF-1R: Phosphorylated insulin-like growth factor 1 receptor
pAKT: Phosphorylated protein kinase B
pS6: Phosphorylated Ribosomal protein S6
Micro-CT: Micro computed tomography
HNSCC: Head and neck squamous cell carcinoma
