# Supplementary material for: IGF-1R mediates crosstalk between nasopharyngeal carcinoma cells and osteoclasts and promotes tumor bone metastasis
Source: J Exp Clin Cancer Res. 2024 Feb 12;43:46. doi: 10.1186/s13046-024-02970-8 (PMC10860326; doi:10.1186/s13046-024-02970-8)
Supplement: Supplementary file 1 — Additional file 1: Supplementary Table 1. Primer sets used for qPCR. Supplementary Table 2. Clinicopathological characteristics of the nasopharyngeal carcinoma sample cohort. Supplementary Table 3. Clinicopathological characteristics of the nasopharyngeal carcinoma study cohort. Supplementary Table 4. Antibodies and reagents used in this study. Supplementary Table 5. The expression of up-regulated genes located in the Plasma membrane were screened. Supplementary Table 6. Gene sets used for GSEA. Supplementary Figure 1. There was no significant difference in survival analysis and in the expression of normal versus tumor tissues of the candidate genes. (a) Survival analysis of candidate genes. (b) Expression of candidate genes in tumor tissues versus normal tissues. (“*” represents P < 0.05). Supplementary Figure 2. Quantitation of IGF-1R immunostaining in paired normal and cancer tissues from the same patient. (“***” represents P < 0.001). Supplementary Figure 3. The protein levels of AKT, pAKT (Thr308), S6 and pS6 (Ser240/244) in 5-8F and 5-8F OE cells cultured in osteoclastic CM. There was no significant difference in the protein levels of pAKT (Thr308) and pS6 (Ser240/244) between the two groups of cells. Supplementary Materials and Methods. [file 13046_2024_2970_MOESM1_ESM.docx]

**Supplementary Table 1** Primer sets used for qPCR.

| **Gene** | **Forward primer (5´-3´)** | **Reverse primer (5´-3´)** |
| --- | --- | --- |
| ***Igf1r*** | TGCTGACCTCTGTTACCTCTCCAC | GTCTTCTCACACATCGGCTTCTCC |
| ***β-actin*** | TCAAGATCATTGCTCCTCCTGA | CTCGTCATACTCCTGCTTGCTG |

**Supplementary Table 2** Clinicopathological characteristics of the nasopharyngeal carcinoma sample cohort.

| **Variables** | **Statistics (N=60)** |
| --- | --- |
| **Age in years** |  |
| N (N miss) | 60 (0) |
| Mean ± SD | 42.67 ± 12.79 |
| **Gender** |  |
| Female | 7 |
| Male | 53 |
| **T Stage** |  |
| T1 | 10 |
| T2 | 17 |
| T3 | 13 |
| T4 | 20 |
| **N Stage** |  |
| N0 | 0 |
| N1 | 22 |
| N2 | 17 |
| N3 | 21 |
| **M Stage** |  |
| M0 | 32 |
| M1 | 28 |

Inclusion Criteria:

1. Age Range: Patients aged between 18 and 70 years.

2. Clinical Diagnosis: Patients diagnosed with nasopharyngeal carcinoma through clinical examination and histological confirmation.

3. Initial Presentation: Patients presenting with the first diagnosis of nasopharyngeal carcinoma, without prior treatment for nasopharyngeal carcinoma.

4. Willingness to Participate: Patients willing and able to provide informed consent, participate in the study, and provide relevant information.

Exclusion Criteria:

1. Prior Treatment for Nasopharyngeal Carcinoma: Patients who have received prior treatment for nasopharyngeal carcinoma before the initial diagnosis, such as surgery, radiation therapy, or chemotherapy.
2. Concurrent Other Cancers: Patients concurrently diagnosed with other types of cancer.
3. Severe Underlying Medical Conditions: Patients with severe underlying medical conditions that may impact study outcomes.
4. Inability to Provide Necessary Information: Patients unable to provide essential information for the study, such as medical history, imaging data, etc.
5. Pregnancy: Female patients who are currently pregnant.

**Supplementary Table 3** Clinicopathological characteristics of the nasopharyngeal carcinoma study cohort.

| **Variables** | **Statistics (N=60)** |
| --- | --- |
| **Age in years** |  |
| N (N miss) | 60 (0) |
| Mean ± SD | 38.28 ± 12.53 |
| **Gender** |  |
| Female | 8 |
| Male | 52 |
| **T Stage** |  |
| T1 | 13 |
| T2 | 14 |
| T3 | 21 |
| T4 | 12 |
| **N Stage** |  |
| N0 | 0 |
| N1 | 25 |
| N2 | 17 |
| N3 | 18 |
| **M Stage** |  |
| M0 | 51 |
| M1 | 9 |

Inclusion Criteria:

1. Age Range: Patients aged between 18 and 70 years.
2. Clinical Diagnosis: Patients diagnosed with nasopharyngeal carcinoma through clinical examination and histological confirmation.
3. Initial Presentation: Patients presenting with the first diagnosis of nasopharyngeal carcinoma, without prior treatment for nasopharyngeal carcinoma.
4. Willingness to Participate: Patients willing and able to provide informed consent, participate in the study, and provide relevant information.

Exclusion Criteria:

1. Prior Treatment for Nasopharyngeal Carcinoma: Patients who have received prior treatment for nasopharyngeal carcinoma before the initial diagnosis, such as surgery, radiation therapy, or chemotherapy.
2. Bone Metastasis: Presence of definitive evidence of bone metastasis at the time of initial diagnosis, as indicated by radiological imaging (such as CT, MRI, bone scan) showing skeletal lesions.
3. Concurrent Other Cancers: Patients concurrently diagnosed with other types of cancer.
4. Severe Underlying Medical Conditions: Patients with severe underlying medical conditions that may impact study outcomes.
5. Inability to Provide Necessary Information: Patients unable to provide essential information for the study, such as medical history, imaging data, etc.
6. Pregnancy: Female patients who are currently pregnant.

**Supplementary Table 4** Antibodies and reagents used in this study.

| List of primary antibodies | | |
| --- | --- | --- |
| Antibody | Source | Catalog Number |
| IGF-1R | Abcam | ab263903 |
| pIGF-1R | Abcam | ab39398 |
| AKT | Cell signaling | #9272 |
| pAKT (Ser473) | Cell signaling | #4060 |
| pAKT (Thr308) | Cell signaling | #9275 |
| S6 | Cell signaling | #2217 |
| pS6 (Ser235/236) | Cell signaling | #2211 |
| pS6 (Ser240/244) | Cell signaling | #2215 |
| GM-CSF | Santa Cruz Biotechnology | sc-32753 |
| IGF-1 | Abcam | ab9572 |
| GAPDH | Fdbio science | FD0063 |

| List of reagents | |
| --- | --- |
| Name | Source |
| Cell Counting Kit-8 | Fdbio science,FD3788 |
| NVP-AEW541 | MedChem Express |
| Rapamycin | Alexis Biochemicals |
| IGF-1 neutralizing antibody | Millipore (05-172) |
| TRAP stain kit | Wako |
| Human GM-CSF ELISA kit | Proteintech, KE00003 |
| Human IGF-1 ELISA kit | Abcam, ab100545 |
| Human IGF-2 ELISA kit | R&D Systems, Bio-Techne, DG200 |
| Mouse IGF-1 ELISA kit | Abcam, ab100695 |
| Mouse IGF-2 ELISA kit | Abcam, ab100696 |
| PrimeScript RT reagent kit | TaKaRa |
| SYBR Premix ExTaq | TaKaRa |
| H&E stain kit | Solarbio |
| GTVisionTM Ⅲ Detection System | Gene Tech |
| RNAprep Pure FFPE Kit | TIANGEN |
| Recombinant human IGF-1 protein | Abcam, ab270062 |
| D-luciferin postassium salt | Beyotime,ST196 |

**Supplementary Table 5** The expression of up-regulated genes located in the Plasma membrane were screened.

| **Location** | **Plasma membrane** | **Location** | **Plasma membrane & Endoplasmic reticulum** |
| --- | --- | --- | --- |
|  | NRCAM |  | RGMA |
|  | IGF-1R |  | SPCS1 |
|  | SMIM36 | **Location** | **Cytosol** |
|  | IVL |  | UBE2D2 |
|  | EREG |  | NRK |
|  | PRKD1 |  | SPRR2F |
| **Location** | **Nucleus** |  | KRTAP10-2 |
|  | NEUROG2 |  | KRT34 |
|  | LBX1 |  | PDCL |
|  | MAFA |  | FBXW9 |
|  | POU6F2 |  | S100A7A |
|  | NKX2-2 |  | WDR81 |
| **Location** | **Extracellular** | **Location** | **Endoplasmic reticulum** |
|  | GAL |  | RCN1 |
|  | DLK1 | **Location** | **Golgi apparatus** |
|  | ARTN |  | HS3ST2 |
|  | PLAT | **Location** | **Unknown** |
|  | GPRC5B |  | ERVV-1 |
|  | CLPSL1 |  | CCDC166 |
| **Location** | **Cytoskeleton** |  |  |
|  | LGALSL |  |  |

**Supplementary Table 6** Gene sets used for GSEA.

Bone mineralization gene list

| **NUM** | **SYMBOL** | **NUM** | **SYMBOL** | **NUM** | **SYMBOL** |
| --- | --- | --- | --- | --- | --- |
| 1 | PHOSPHO1 | 13 | COL1A2 | 25 | BGLAP |
| 2 | LOX | 14 | LEP | 26 | FGR |
| 3 | ASPN | 15 | PKDCC | 27 | PRICKLE1 |
| 4 | ZBTB40 | 16 | RSPO2 | 28 | SLC24A3 |
| 5 | BMP2 | 17 | SOX9 | 29 | FGFR2 |
| 6 | ERCC2 | 18 | FGFR3 | 30 | GPC3 |
| 7 | ANKH | 19 | TUFT1 | 31 | FBXL15 |
| 8 | LTBP3 | 20 | EIF2AK3 | 32 | IFT80 |
| 9 | PTHLH | 21 | PTN | 33 | ALOX15 |
| 10 | KLF10 | 22 | PTH1R | 34 | PTH |
| 11 | SNX10 | 23 | HIF1A |  |  |
| 12 | IBSP | 24 | COMP |  |  |

Bone morphogenesis gene list

| **NUM** | **SYMBOL** | **NUM** | **SYMBOL** | **NUM** | **SYMBOL** |
| --- | --- | --- | --- | --- | --- |
| 1 | GHR | 15 | IFITM5 | 29 | LTF |
| 2 | GLI3 | 16 | NPPC | 30 | CITED2 |
| 3 | ATF2 | 17 | CER1 | 31 | OSR2 |
| 4 | FGF4 | 18 | ATG9A | 32 | SERPINH1 |
| 5 | HOXA11 | 19 | ACP5 | 33 | NEUROG1 |
| 6 | RIPPLY2 | 20 | AXIN2 | 34 | IHH |
| 7 | MSX2 | 21 | TMEM119 | 35 | HAS2 |
| 8 | RARG | 22 | TMEM107 | 36 | ANXA6 |
| 9 | EXT1 | 23 | FOXN3 | 37 | STC1 |
| 10 | SMPD3 | 24 | MATN1 | 38 | FGFR2 |
| 11 | LRP5 | 25 | TSKU | 39 | CDX1 |
| 12 | SFRP4 | 26 | MMP13 | 40 | ALPL |
| 13 | MEGF8 | 27 | FOXC1 | 41 | PEX7 |
| 14 | FGF18 | 28 | SOX9 |  |  |


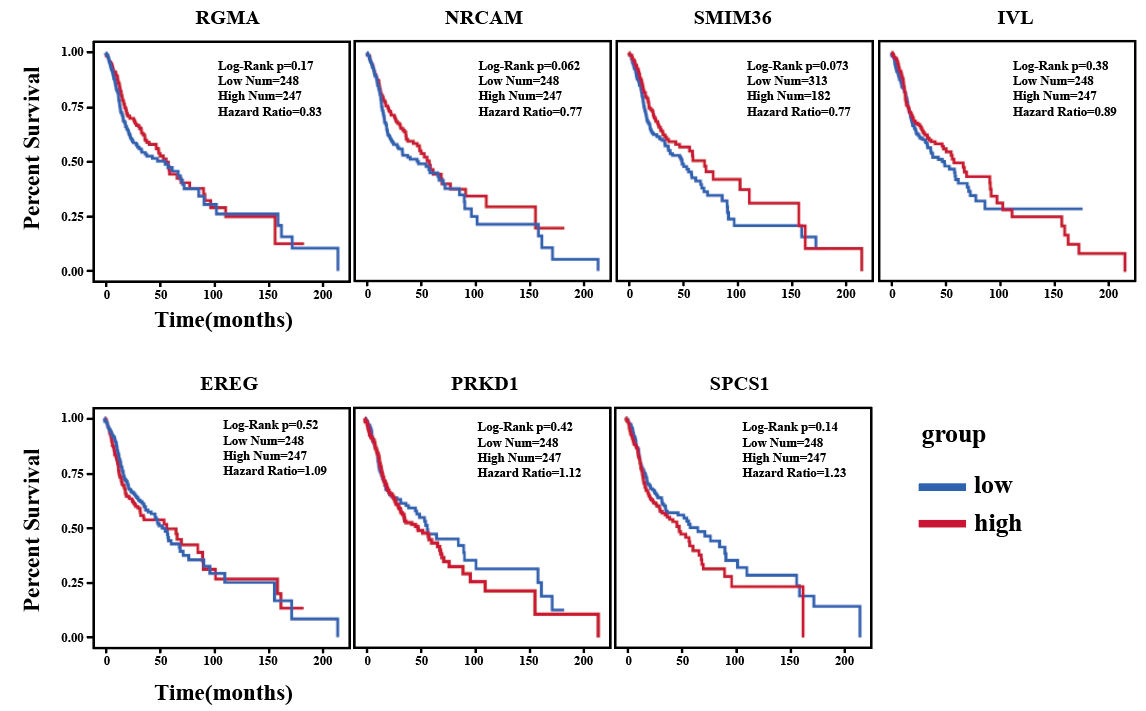

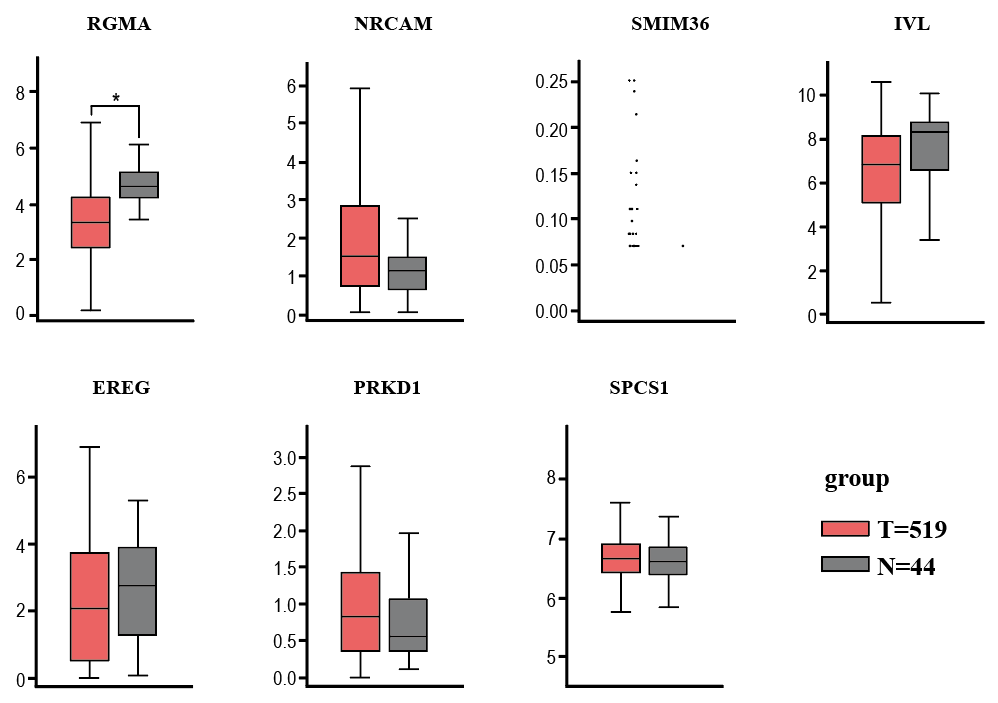


**b**

**a**

**Supplementary Figure 1 There was no significant difference in survival analysis and in the expression of normal versus tumor tissues of the candidate genes.** (a) Survival analysis of candidate genes. (b) Expression of candidate genes in tumor tissues versus normal tissues. (“*” represents P < 0.05.)

**
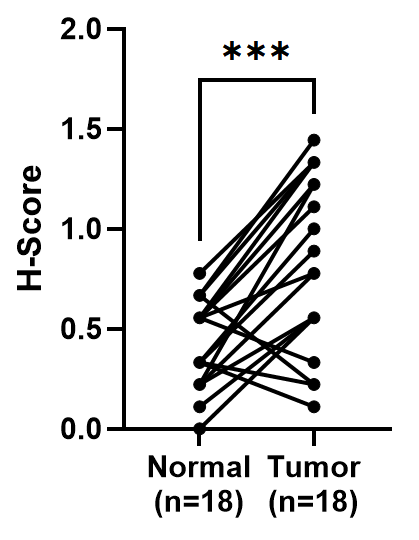
**

**Supplementary Figure 2** Quantitation of IGF-1R immunostaining in paired normal and cancer tissues from the same patient. (“***” represents P < 0.001.)


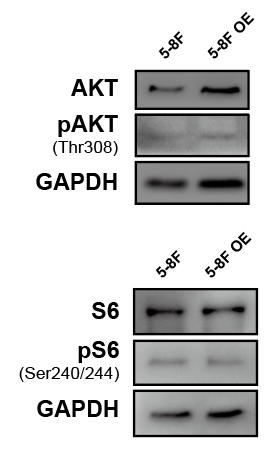


**Supplementary Figure 3** The protein levels of AKT, pAKT (Thr308), S6 and pS6 (Ser240/244) in 5-8F and 5-8F OE cells cultured in osteoclastic CM. There was no significant difference in the protein levels of pAKT (Thr308) and pS6 (Ser240/244) between the two groups of cells.

**Supplementary Materials and Methods**

**Establishment of the BM3 bone metastasis cell line.** The 5-8F parental cells with stable expression of firefly luciferase (LUC) were trypsinised and suspended in ice-cold PBS at a density of 4×10^6^/mL. Male BALB/c nude mice (3-4 weeks old) were anaesthetised with 2% sodium pentobarbital and fixed in the ventral position, with the skin disinfected with 70% ethanol. 200 μL cell suspension was injected into the left ventricle from the left sternal angle using an insulin syringe (32G) within 1 minute. When the needle was successfully inserted into the left ventricle, a distinct pulse of bright red blood should appear in the syringe. After slowly injecting the cells, the plunger is pulled upwards slightly to create a small amount of negative pressure and reduce the drip of cells into the thoracic cavity. After removing the needle, apply gentle pressure to the chest at the injection site to minimise bleeding. The mice were moved to heating pad until fully awake. The formed bone metastatic tumors were monitored by BLI. When stable bone metastatic lesions were monitored, mice were euthanised, tissues were isolated to obtain metastatic lesions (usually distal femur), bones were minced and incubated in collagenase digesting solution at 37℃ and the cells were collected by centrifugation and seeded onto culture dishes containing RPMI-1640 medium with 10% FBS. The medium was replaced after 48 hours to eliminate non-adherent cells, and this obtained cell subpopulation, was expanded in culture, and then re-injected intracardially into mice for the next round. In the first round, ten mice were injected with 5-8F cells, two of which developed bone metastasis, and cells were collected from the site of the bone metastasis named BM1. In the second round, eight mice were injected with BM1, three of which developed bone metastasis, and the cells were collected named BM2. In the third round, eight mice were injected with BM2, five of which developed bone metastasis, and the cells were collected named BM3. The cells collected of each round were cultured in vitro for 10 to 18 days, depending on their growth, and then injected back into the mice. Mice injected intracardially with BM3 cells showed strong metastatic signals only in the bones but not in the lungs and/or liver at about two weeks (Figure 1a).
